# Traumatic Spinal Subarachnoid Hemorrhage With Spinal Cord Compression: A Case Report and Literature Review

**DOI:** 10.7759/cureus.69005

**Published:** 2024-09-09

**Authors:** Melanie Buchta, Albert Eckert, Christoph J. Griessenauer, Lukas Grassner

**Affiliations:** 1 Department of Neurosurgery, University Hospital Salzburg, Paracelsus Medical University, Salzburg, AUT; 2 Department of Neurosurgery, Christian Doppler Medical Center, Paracelsus Medical University, Salzburg, AUT

**Keywords:** case report, spinal cord compression, spinal subarachnoid hemorrhage, surgical decompression, traumatic

## Abstract

The occurrence of spinal hematoma is rare, and differentiation between subarachnoid hemorrhage and subdural hemorrhage on neuroradiological imaging can be challenging. Spinal subarachnoid hemorrhage (SSAH) is less frequently associated with trauma and can result in severe neurological impairment.

We report the case of a 53-year-old man who presented with severe motor and sensory deficits primarily of the left arm without further neurological deficits in the other extremities after a fall from a height of six meters. Magnetic resonance imaging (MRI) showed an acute intradural hematoma at the C4-C6 level with significant spinal cord compression. Surgery revealed a hematoma enclosed by an arachnoid layer. Two months later, MRI showed complete resolution of spinal cord edema and avulsion of the left C6 and partially C7 nerve roots, corresponding to electromyography findings revealing a brachial plexus avulsion. Nine months after the accident and five months after surgical repair of the brachial plexus avulsion, there was a significant improvement in the sensory and motor functions of the left arm, allowing the patient to manage daily activities independently.

Patients with neurological deficits following spinal trauma should be evaluated for spinal cord compression, such as intraspinal hematoma, as soon as possible to enable early spinal decompression. We describe a rare case of traumatic SSAH and brachial plexus avulsion following successful surgical decompression of the spinal cord without clinical postoperative myelopathy.

## Introduction

Spinal hematoma is a rare phenomenon that may cause serious neurologic symptoms [1]. They can be classified as spinal epidural hemorrhage, spinal subdural hemorrhage (SSDH), or spinal subarachnoid hemorrhage (SSAH) based on the location of the bleeding [2]. Approximately 15% of all spinal hematomas are located within the subarachnoid space [1]. Plotkin et al. first described the macroscopic features and surgical removal of the extremely rare SSAH as a clot beneath the intact arachnoid [3]. Differentiating between SSAH and SSDH using neuroradiologic imaging, such as magnetic resonance imaging (MRI) and computed tomography (CT), is difficult, and diagnosis often relies on surgery or autopsy [4]. SSAH may be associated with hemorrhagic disorders [5], anticoagulation therapy [6], rupture of arteriovenous malformations or aneurysms [1], or less commonly after traumatic events.

To the best of our knowledge, researchers have reported only 12 cases of SSAH after spinal trauma [4-15]. We report the case of a 53-year-old man with traumatic SSAH and brachial plexus avulsion after a fall, causing severe motor deficits and hypesthesia of the left arm without further neurological deficits in other extremities.

## Case presentation

**Figure 1:**
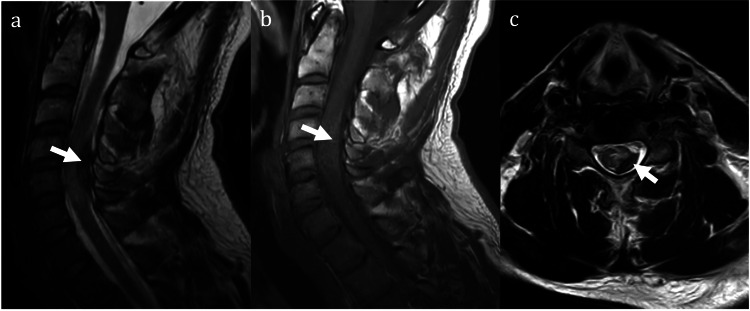
Preoperative MRI images showing traumatic spinal intradural hemorrhage The sagittal T2-weighted image shows a dorsal hypointense fusiform bleeding from C4 to C6 level with associated cord edema (a), in the sagittal T1-weighted image, the bleeding displayed isointense (b) and the axial T2-weighted image shows hypointense bleeding located in the right dorsolateral aspect of the spinal cord with central cord edema (c).

A 53-year-old previously healthy male patient fell from a height of six meters and presented approximately one hour after the accident to the emergency department of a community hospital. Physical examination revealed severe sensory and motor deficits in the left upper extremity, with loss of sensation in the left arm except for dermatome C8, and residual motor strength in finger flexion and adduction graded as 2/5, as well as ulnar-sided finger flexion and abduction graded as 3/5. Despite this, there was no motor function in the rest of the left arm. No further motor or sensory deficits were found in the other extremities. Laboratory tests including coagulation profile and vital parameters were within normal limits. Chest CT scan revealed bilateral pneumothorax, multiple rip fractures, left-sided clavicle and scapula fractures, and multiple minor thoracic vertebral body compression fractures. CT angiography and bone scan of the cervical spine, performed without a native sequence, did not reveal any traumatic dissection of the cervical vessels or fractures of the cervical spine. A cervical spine MRI was performed the following day and revealed an intradural hematoma at the C4-C6 level with significant left dorsolateral spinal cord compression (Figure 1). The hematoma was isointense on T1-weighted images and hypo- to hyperintense on T2-weighted images, consistent with an acute hematoma. In addition, a brachial plexus MRI was performed without definite exclusion of a brachial plexus injury due to extensive soft tissue swelling. The patient was then admitted to the medical intensive care unit of the community hospital pending neurological, orthopedic, and neurosurgical consultation. Two bilateral pleural drains were placed, and the patient was transferred to the operating room of our neurosurgical department approximately 38 hours after the accident, as the community hospital lacked the specialization for treating spinal injuries. We maintained the mean arterial pressure at 85-90 mm Hg and administered dexamethasone.

**Figure 2:**
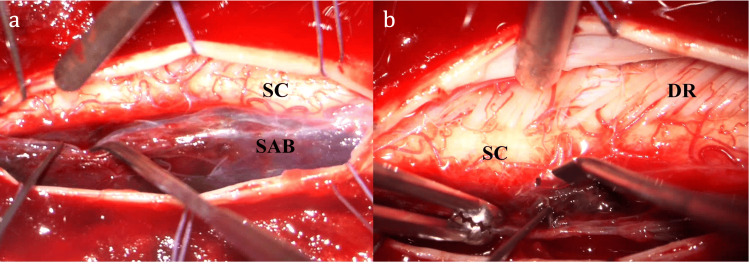
Intraoperative images after dural opening The left-sided hematoma is covered by arachnoid and displaces the spinal cord laterally (a). A careful opening of the arachnoid is implemented. After removal of the hematoma, a hyperemia of the left sided cord is visible (b). SC: spinal cord, SAB: subarachnoid hemorrhage, DR: dorsal roots

We performed a microsurgical laminectomy at the C4 to C6 level and found a thin epidural hematoma. Intraoperative ultrasound showed an intradural extramedullary hematoma that significantly displaced the spinal cord. We performed a midline durotomy revealing the hematoma surrounded by an arachnoid layer (Figure 2a). We then carefully opened the arachnoid with a sapphire blade and completely evacuated the blood clot using suction and microsurgical dissection until we could see the underlying neurological structures clearly (Figure 2b). Inspection showed hyperemia of the spinal cord parenchyma without active bleeding, vascular malformation, or tumor lesion. We closed the dura with a running suture, covered it with a sponge sealant patch, and completed the procedure with a C3 to C6 spinal fusion using lateral mass screws and rod placement.

**Figure 3:**
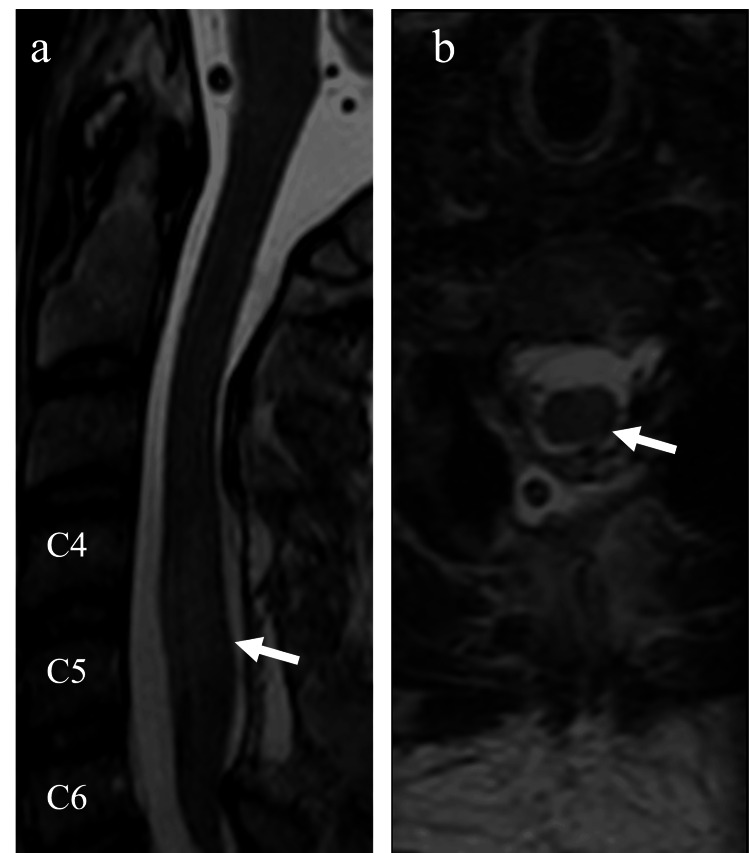
Postoperative MRI Sagittal T2-weighted image (a) and axial T2-weighted image (b) showing a sufficient decompression of the spinal canal with spinal cord edema (arrow) from C4 to C6 level. There is no residual hemorrhage visible (a, b).

Immediate postoperative neurological examination was unchanged. An MRI two days after surgery showed adequate decompression of the spinal canal with mild spinal cord edema and no residual intradural hematoma (Figure 3). 

A follow-up MRI two months later showed complete resolution of spinal cord edema and avulsion of the left C6 and partially C7 nerve roots, consistent with electromyographic findings showing a left-sided brachial plexus avulsion. Neurological evaluation revealed left-sided improvement in finger flexion and adduction with a strength grade of 3/5 and 4/5 for ulnar-sided finger flexion and abduction. Additionally, the patient developed new dysaesthesia in the left C6 dermatome. At another follow-up after four months, just prior to a brachial plexus surgery, the patient presented with a left shoulder depression and internal rotation of the arm. We observed an improvement of hypesthesia in the thenar region as well as the dorsal side of the middle finger and regression of the dysaesthesia. Arm adduction, internal rotation and anteversion were graded as 4/5 in strength. We also noted improvement in distal motor function, with wrist and finger flexion as well as finger adduction graded as 4-5/5. Finger abduction was graded as 2-3/5, except for the abductor digiti minimi muscle, which was graded as 5/5.

Colleagues from the plastic surgery department performed an autologous transplantation and nerve transfer of the ipsilateral C5 nerve root with a sural nerve interposition on the axillary nerve and the radial nerve. In addition, they carried out a hemi-dorsal scapular nerve transfer with sural interposition to the suprascapular nerve, a nerve transfer of wrist flexor fascicular parts of the ulnar nerve directly to the biceps branch of the musculocutaneous nerve, and a nerve transfer of wrist parts of the median nerve to the brachial branch of the musculocutaneous nerve. Approximately four months after brachial plexus surgery and two months of rehabilitation and physiotherapy with regular electrical stimulation therapy, we observed an improvement in triceps function with a muscle strength level of 3/5. We also noted a slight improvement in elbow flexion graded as 1/5 and a regressive shoulder depression. The patient reports no complaints regarding the cervical spine, only a slight restriction in ante- and retroflexion. X-rays reveal no evidence of misalignment or instability of the cervical spine. The patient, who works as a veterinarian, is already able to perform the majority of his original professional tasks and requires only minimal assistance. 

## Discussion

SSAH following a traumatic event to the spine is extremely rare. We describe the case of a patient with SSAH after a fall presenting with severe motor and sensory deficits of the left arm, with subsequent surgical evacuation of the hematoma and decompression of the spinal cord. The pathophysiology of traumatic SSAH is not completely understood. It is thought to result from rupture of radicular blood vessels within the subarachnoid space due to a sudden increase in abdominal and thoracic pressure, which increases intravascular pressure secondary to trauma [16]. Russell et al. report a case of SSAH resulting from avulsion of cervical nerve roots [12], which was a probable mechanism in our case. A clot is then formed by blood collection, causing compression of the spinal cord and nerve roots. Commonly, the continuous diluting effect of the cerebrospinal fluid (CSF) prevents the formation of a blood clot. Severe hemorrhage or reduced CSF flow in the relevant subarachnoid compartment may be responsible for interfering with this process [5].

Neuroradiologic diagnosis of SSAH can be challenging, with the majority of cases, including ours, being diagnosed based on surgical exploration or autopsy findings. Frager et al. identified a characteristic myelographic aspect and described a filling effect called "capping" [17]. Domenicucci et al. identified SSAH on CT and MRI because the blood clot was surrounded by CSF and separated from the dura mater. Therefore, the hematoma could be distinguished from SSDH [5]. A typical blood collection of SSDH is called the "inverted Mercedes-Benz sign" due to its configuration with two lateral denticulate ligaments and the midline dorsal septum creating three collecting subdural compartments [18].

To the best of our knowledge, researchers have published only 12 cases of SSAH after spinal cord trauma in the English literature. The clinical manifestation in most cases was hemiparesis, paraparesis, or Brown-Séquard syndrome. Many of the described cases exhibited neurological symptoms only after several hours or even days, leading to subsequent surgical intervention. This frequently resulted in either partial recovery or no improvement (Table 1). Ultimately, early detection of traumatic SSAB and prompt surgical intervention could have prevented neurological deficits. In contrast, one case demonstrated spontaneous remission after a few days with conservative management [6]. In our case, the patient suffered from brachial plexus avulsion but showed no signs of clinical myelopathy. However, MRI showed signal alterations in the spinal cord at the site of the hematoma, indicating spinal cord injury. Surgical decompression should be performed after spinal cord trauma to minimize secondary injury events. The STASCIS trial showed a better neurological outcome when surgery was performed within 24 hours of the traumatic event and some studies recommend even earlier decompression [19]. We maintained a mean arterial pressure of 85-90 mm Hg just prior to surgery and for an additional five days afterward. We performed surgery within 24 hours but approximately 38 hours after the accident, due to the delayed presentation by the community hospital, which did not have a spine center.

**Table 1:** Literature review of cases with traumatic spinal subarachnoid hematoma F: Female, M: Male, yrs: years, N/A: not available, *the time in hours is referenced from the point of hospital admission, **the time in hours is referenced from the time of the accident

Authors and Year	Age (yrs)	Gender	Level	Cause	Clinical presentation	symptom onset	Treatment	Recovery
de Andrada Pereira et al., 2022 [8]	83	F	C3-5	Domestic fall	Brown-Séquard	~ 8 h*	Laminectomy and fusion	No
Di Rienzo et al., 2013 [9]	71	M	C1-3	Vehicular accident	Hemiparesis	~ 24 h*	Laminectomy and duraplasty	Partial
Domenicucci et al., 2005 [5]	70	F	C2-4	Stair fall	Neck pain	N/A	Conservative	Good
Mori et al., 1987 [10]	43	M	C2-3	Hyperextension cervical trauma	Brown-Séquard	N/A	Operative unspecified	Partial
Rascón-Ramirez et al., 2018 [11]	83	F	C3-5	Stair fall	Brown-Séquard	N/A	Hemilaminectomy	Partial
Russell et al., 1980 [12]	19	M	C1-6	Hyperextension cervical trauma	Quadriplegia	N/A	Laminectomy	No
Wolfe et al., 2017 [6]	67	M	C6-7	Domestic fall	Brown-Séquard	~ 24 h*	Conservative	Good
Jang et al., 2005 [13]	66	M	Th11-12	Fall from a height of 3 m	Paraplegia	~ 3 h*	Hemilaminectomy	No, death
Chang et al., 2012 [7]	63	F	C1	Domestic fall	Quadriparesis	~ 72 h**	Laminectomy	No
Kim et al., 2015 [14]	17	M	C4-5	Bike accident	Hemiparesis	~ 8-12 h*	Laminectomy	Partial
Gupta et al., 1997 [4]	7	M	Th10-L2	Vehicular accident	Paraplegia	immediate	Laminectomy	No
Lee et al., 2009 [15]	78	F	L1-2	Vehicular accident	Paraparesis	immediate	Laminectomy and fusion	Partial
Present Case	53	M	C4-6	Fall from a height of 6 m	Monoparesis	immediate	Laminectomy and fusion	Partial

We then performed surgical laminectomy for dural exposure and hematoma removal followed by spinal fusion. The patient did not have an absolute indication for dorsal stabilization of the cervical spine, as no unstable bone fractures were present. Ultimately, a laminectomy alone could have been performed. However, due to the need for a multi-level laminectomy, the surgeon's expertise led to the decision to perform a cervical fusion. Moreover, evidence supports spinal fusion following multilevel laminectomy to prevent severe deformities, such as post-laminectomy kyphosis [20]. In our literature review, most patients underwent decompression surgery without spinal fusion and only two patients were managed conservatively. The outcome was variable with mostly partial improvement depending on the initial neurological deficits, duration of spinal cord compression and clot volume (Table 1). 

## Conclusions

MRI of the spine should be performed promptly in patients after traumatic spinal injury. Differentiation between SSAH and SSDH is usually inconclusive and often relies on surgical exploration. Surgeons should perform decompression of the spinal canal when significant cord compression is detected to prevent secondary injury events. Nevertheless, the lack of evidence in these cases necessitates further research. Combined with surgical repair of the brachial plexus avulsion and evacuation of the SSAB, followed by spinal fusion, the patient experienced significant improvement in motor and sensory function of the left arm, leading to independence in daily activities.
